# Prognostic Capability of Clinical SYNTAX Score in Patients with Complex Coronary Artery Disease and Chronic Renal Insufficiency Undergoing Percutaneous Coronary Intervention

**DOI:** 10.31083/j.rcm2501018

**Published:** 2024-01-10

**Authors:** Mengyao Li, Xu Liu, Mao Jiang, Yumeng Lei, Zhongpei Li, Shicheng Li, Ying Mao, Xufen Cao, Liqiu Yan

**Affiliations:** ^1^Department of Cardiology & Dongguan Cardiovascular Research Institute, Dongguan Songshan Lake Central Hospital, Guangdong Medical University, 523326 Dongguan, Guangdong, China; ^2^Department of Cardiology, Cangzhou Central Hospital, Hebei Medical University, 061017 Cangzhou, Hebei, China

**Keywords:** complex coronary artery disease, chronic renal insufficiency, clinical SYNTAX score, SYNTAX score, percutaneous coronary intervention

## Abstract

**Background:**

The SYNTAX score (SS) is useful for predicting clinical outcomes in patients undergoing percutaneous coronary intervention (PCI). The clinical SYNTAX score (CSS), developed by combining clinical parameters with the SS, enhances the risk model’s ability to predict clinical outcomes. However, prior research has not yet evaluated the prognostic capacity of CSS in patients with complex coronary artery disease (CAD) and chronic renal insufficiency (CRI) who are undergoing PCI. We aimed to demonstrate the prognostic potential of CSS in assessing long-term adverse events in this high-risk patient cohort.

**Methods:**

A total of 962 patients with left main and/or three-vessel CAD and CRI were enrolled in the study spanning from January 2014 to September 2017. The CSS was calculated by multiplying the SS by the modified age, creatinine, and left ventricular ejection fraction (ACEF) score (age/ejection fraction + 1 for each 10 mL of creatinine clearance <60 mL/min per 1.73 $m^{2}$). The patients were categorized into three groups based on their CSS values: low-CSS group (CSS <18.0, n = 321), mid-CSS group (18.0 ≤ CSS < 28.3, n = 317), and high-CSS group (CSS ≥28.3, n = 324) as per the tertiles of CSS. The primary endpoints were all-cause mortality (ACM) and cardiac mortality (CM). The secondary endpoints included myocardial infarction (MI), unplanned revascularization, stroke, and major adverse cardiac and cerebrovascular events (MACCE).

**Results:**

At the median 3-year follow-up, the high-CSS group exhibited higher rates of ACM (19.4% vs. 6.6% vs. 3.6%, *p* < 0.001), CM (15.6% vs. 5.1% vs. 3.2%, *p* = 0.003), and MACCE (33.8% vs. 29.0% vs. 20.0%, *p* = 0.005) in comparison to the low and mid-CSS groups. Multivariable Cox regression analysis revealed that CSS was an independent predictor for all primary and secondary endpoints (*p* < 0 .05). Moreover, the C-statistics of CSS for ACM (0.666 vs. 0.597, *p* = 0.021) and CM (0.668 vs. 0.592, *p* = 0.039) were significantly higher than those of SS.

**Conclusions:**

The clinical SYNTAX score substantially enhanced the prediction of median 3-year ACM and CM in comparison with SS in complex CAD and CRI patients following PCI.

## 1. Introduction 

Cardiovascular disease (CVD) and chronic renal insufficiency (CRI) are global public health concerns [1]. Earlier research has indicated an increasing prevalence of concomitant CVD with worsening renal function [2]. Patients with CRI have a lower success rate, higher risk of complications, and worse clinical results in comparison with normal renal function patients while receiving percutaneous coronary intervention (PCI) [3]. Therefore, identifying high-risk CRI patients and undertaking early warning and intervention measures could enhance the clinical results following PCI. The SYNTAX score (SS) is recognized as a vital tool for guiding decisions between coronary artery bypass grafting (CABG) and PCI [4, 5, 6]. It has been demonstrated that the SS’s utility in objectively selecting the most appropriate revascularization technique can be further enhanced by the inclusion of clinical factors [7]. The clinical SYNTAX score (CSS), calculated by multiplying the SS with the modified age, creatinine, and left ventricular ejection fraction (ACEF) score (${\mathrm{ACEF}}_{\text{CrCl}}$ Score: age/ejection fraction + 1 for each 10 mL the creatinine clearance <60 mL/min per 1.73 $m^{2}$), has been validated for accurately predicting long-term adverse event risks in patients undergoing PCI [8, 9]. However, no study has yet investigated the predictive ability of the CSS for long-term clinical outcomes in patients with complex CAD and CRI following PCI.

## 2. Methods

### 2.1 Study Subjects

**Fig. 1.**
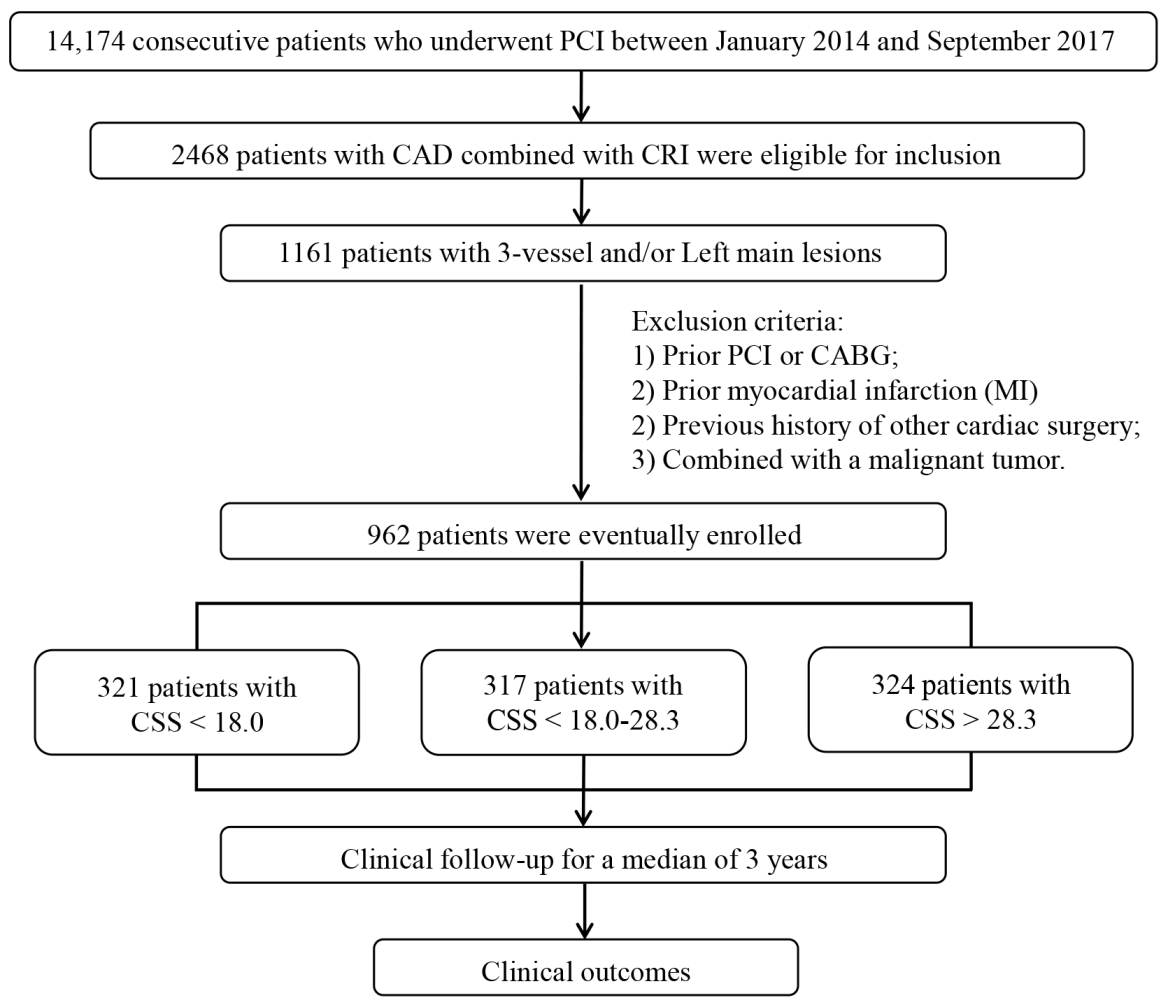
**Flow chart of this study**. CAD, coronary artery disease; CRI, chronic renal insufficiency; CABG, coronary artery bypass grafting; PCI, percutaneous coronary intervention; MI, Myocardial infarction; CSS, Clinical SYNTAX score.

In total 14,174 patients who underwent PCI in Cangzhou Central Hospital, Hebei Medical University from January 2014 to September 2017 were retrospectively enrolled. The glomerular filtration rate was estimated for all patients utilizing the simplified Modification of Diet in Renal Disease method. As per our previous description, a subset of 2468 patients exhibited an assessed glomerular filtration rate (eGFR) of <90 mL/min/1.73 $m^{2}$ [10, 11]. Among them, 1161 patients with left main and/or three-vessel CAD were present. The study employed the following exclusion criteria: (1) Prior PCI or CABG; (2) Prior myocardial infarction (MI); (3) Previous history of other cardiac surgery; (4) Combined with a malignant tumor. Eventually, a cohort of 962 patients diagnosed with left main disease and/or triple-vessel CAD and CRI were included in the study. All patients were stratified into three groups based on CSS tertiles: group with low CSS (CSS <18.0, n = 321), group with mid-CSS (18.0 ≤ CSS < 28.3, n = 317), and group with high CSS (CSS ≥28.3, n = 324) (Fig. 1). The Institutional Review Board of Cangzhou Central Hospital, Hebei Medical University granted its approval for the research procedures. This research adheres to the guidelines delineated in the Declaration of Helsinki. Before the intervention, each patient provided written informed consent.

### 2.2 Clinical SYNTAX Score

As per the website’s description (https://syntaxscore.org/), the SS for every patient was calculated for each lesion with ≥50% diameter stenosis in vessels with a >1.5 mm lumen [4]. The coronary angiograms underwent independent adjudication by two of three experienced cardiologists. To resolve any disagreements, a third cardiologist was consulted. Each patient’s CSS was computed employing the following equation: CSS = ${\mathrm{ACEF}}_{\text{CrCL}}$× SS. The ${\mathrm{ACEF}}_{\text{CrCL}}$ was computed employing the formula below: age/ejection fraction +1 for each 10 mL/min the creatinine clearance <60 mL/min per 1.73 $m^{2}$ (up to a maximum of 6 points). Consequently, individuals with a CrCl between 50 to 59 mL/min per 1.73 $m^{2}$, 40 to 49 mL/min per 1.73 $m^{2}$, and 30 to 39 mL/min per 1.73 $m^{2}$ would receive 1, 2, and 3 points, respectively [8].

### 2.3 Follow-Up and Outcomes

All patients who were enrolled underwent annual follow-up conducted through outpatient visits or telephone interviews. All-cause mortality (ACM) and cardiac mortality (CM) were the primary endpoints. Secondary endpoints comprised of unplanned revascularization, MI, stroke, and major adverse cardiac and cerebrovascular events (MACCE). ACM, unplanned revascularization, MI, and stroke were all integrated to define MACCE. Unless a non-cardiogenic cause was established, all deaths were attributed to CM. The fourth universal definition of MI served as the criterion for defining MI [12].

### 2.4 Statistical Analysis

R software version 3.6.0 and SPSS version 24.0 (IBM Corp., Armonk, NY, USA) were employed for the statistical analyses. The continuous variables were presented as mean ± standard deviation (SD) or median (inter-quartile range), based on normality. Utilizing the Kolmogorov-Smirnov test, these variables were assessed, and the Kruskal Wallis H test or one-way-Analysis of Variance was conducted for the comparison. Fisher’s exact test or chi-square test was utilized to compare the categorical variables, which were presented as frequency (percentage). The Kaplan-Meier cumulative risk curves were presented as per the CSS groups and the log-rank test was employed to make a comparison. Utilizing the enter method, Cox proportional hazards regression was employed to perform the multivariate survival analysis for median 3-year clinical outcomes. The area under the curve (AUC) values for the receiver operator characteristic (ROC) curves were utilized to compare the prognosis predictive accuracy of the SS, modified ACEF score, and CSS for clinical results at a median 3-year follow-up. The consistency between the observed and predicted risks was evaluated with calibration plots and the Hosmer-Lemeshow test [13]. For all tests, a two-sided *p*-value < 0.05 was deemed statistically significant.

## 3. Results

### 3.1 Baseline Features

The baseline clinical, angiographic, and procedural features of patients, stratified based on the tertiles of CSS, are presented in Table 1 and **Supplementary Table 1**. The median age of patients was 66.0 (60.0–71.0) years, and the study encompassed 559 (58.1%) male patients. The SS ranged from 5.0 to 44.5, while the modified ACEF score spanned from 0.55 to 2.38. The range of CSS was 5.7 to 184.2. As compared to the patients of the group with low or mid-CSS, those in the group with high CSS were older (*p* < 0.001), had a greater likelihood of having a diabetes history (*p* = 0.018), previous stroke (*p* = 0.042), worse renal function (*p* < 0.001) and left ventricular ejection fraction (LVEF) (*p* < 0.001), and exhibited more complex angiographic features, like heavy calcification, tortuosity, bifurcation, and diffuse lesions (*p* < 0.001 for all).

**Table 1.** **Baseline clinical features of patients**.

		CSS <18.0	CSS <18.0–28.3	CSS >28.3	*p*-value
		(n = 321)	(n = 317)	(n = 324)	
Age, years		63.0 (57.0–68.0)	66.0 (61.0–69.0)	69.0 (64.0–73.8)	<0.001
Sex					0.596
	Female	128 (39.9)	133 (42.0)	142 (43.8)	
	Male	193 (60.1)	184 (58.0)	182 (56.2)	
BMI, ${\mathrm{kg/m}}^{2}$		26.09 ± 3.15	26.02 ± 3.06	26.35 ± 3.32	0.381
Hypertension		224 (69.8)	226 (71.3)	224 (69.1)	0.830
Diabetes		66 (20.6)	79 (24.9)	98 (30.2)	0.018
Hyperlipidemia		124 (38.6%)	128 (40.4)	139 (42.9)	0.540
Previous Smoking		37 (11.5)	47 (14.8)	28 (8.6)	0.051
Previous Stroke		24 (7.5)	34 (10.7)	44 (13.6)	0.042
COPD, n (%)		5 (1.6)	2 (0.6)	9 (2.8)	0.103
eGFR, mL/min		81.0 (73.9–85.8)	79.5 (72.4–85.5)	70.8 (54.6–80.9)	<0.001
Renal function					<0.001
	60 ≤ eGFR < 90	319 (99.4)	307 (96.8)	207 (63.9)	
	30 ≤ eGFR < 60	2 (0.6)	10 (3.2)	104 (32.1)	
	eGFR <30	0 (0)	0 (0)	13 (4)	
Heart function					0.371
	I	264 (82.2)	273 (86.1)	268 (82.7)	
	II	42 (13.1)	30 (9.5)	36 (11.1)	
	III	6 (1.9)	8 (2.5)	14 (4.3)	
	IV	9 (2.8)	6 (1.9)	6 (1.9)	
LVEF, %		63.6 (60.0–67.9)	62.0 (56.9–66.0)	60.0 (51.0–64.2)	<0.001
LVEDD (mm)		47.1 (45.0–52.7)	47.4 (44.9–51.0)	47.0 (44.1–50.3)	0.018
Creatinine (mg/dL)		0.9 (0.8–1.0)	0.9 (0.8–1.0)	1.0 (0.9–1.3)	<0.001

Values are mean ± SD, median (IQR), or n (%). CSS, clinical SYNTAX score; BMI, body mass index; COPD, chronic obstructive pulmonary disease; eGFR, estimated glomerular filtration rate; LVEF, left ventricular ejection fraction; LVEDD, left ventricular end-diastolic diameter.

### 3.2 Clinical Results

**Fig. 2.**
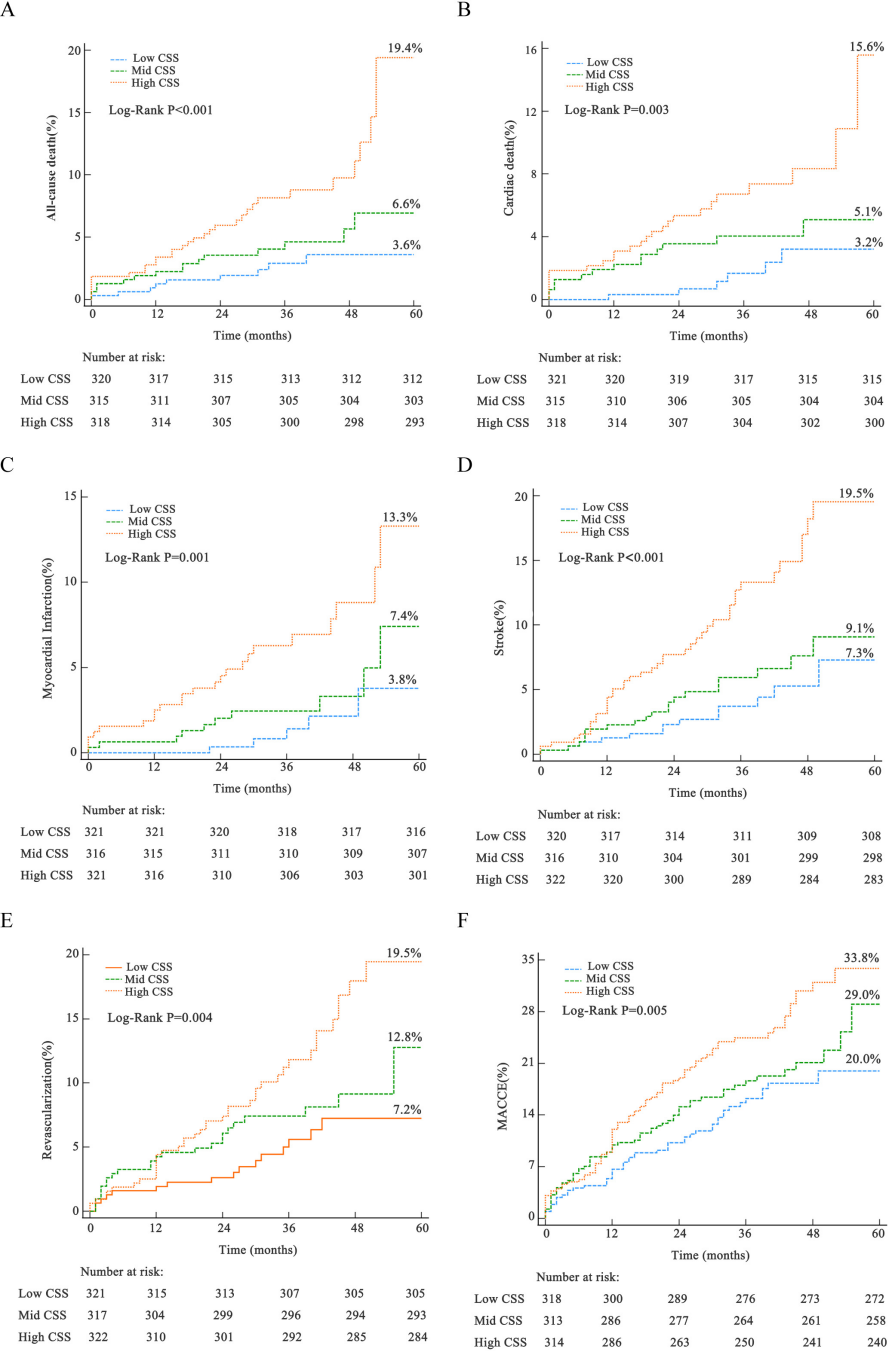
**Event rates depicted by Kaplan-Meier curves, stratified by CSS across five years**. (A) All-cause death. (B) Cardiac death. (C) Myocardial infarction. (D) Stroke. (E) Unplanned revascularization. (F) Major adverse cardiovascular and cerebrovascular events. MACCE, major adverse cardiac and cerebrovascular events; CSS, clinical SYNTAX score.

The cumulative rates of adverse events over a 5-year period, stratified as per the tertiles of CSS, have been presented in Fig. 2 and **Supplementary Table 2**. The Kaplan-Meier cumulative risk curves demonstrated that the high CSS group exhibited the highest incidences of ACM (19.4% vs. 6.6% vs. 3.6%, *p* < 0.001), CM (15.6% vs. 5.1% vs. 3.2%, *p* = 0.003), MI (13.3% vs. 7.4% vs. 3.8%, *p* = 0.001), unplanned revascularization (19.5% vs. 12.8% vs. 7.2%, *p* = 0.004), stroke (19.5% vs. 9.1% vs. 7.3%, *p* < 0.001), and MACCE (33.8% vs. 29.0% vs. 20.0%, *p* = 0.005) among the three groups.

### 3.3 Multivariable Cox Regression Analysis

**Fig. 3.**
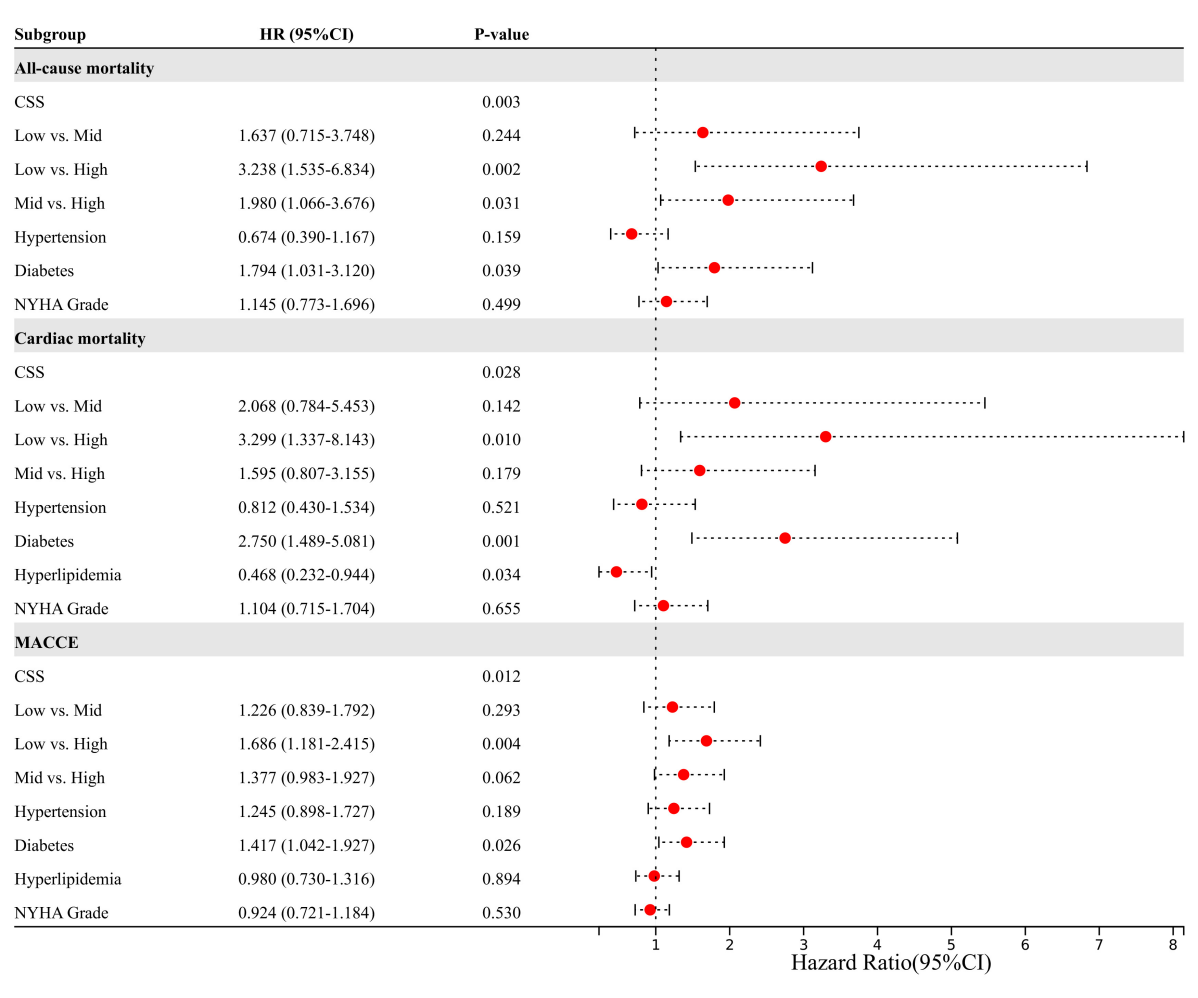
**Multivariate Cox proportional hazards regression for all-cause mortality, cardiac mortality and major adverse cardiovascular and cerebrovascular events**. MACCE, major adverse cardiac and cerebrovascular events; CSS, clinical SYNTAX score; NYHA, New York Heart Association; HR, hazard ratio.

In terms of ACM, the univariable cox regression analysis indicated that the high CSS group exhibited an expected 3.485-fold and 2.075-fold increase in risk compared to the low and medium CSS group, respectively (all *p* < 0.05). However, the CSS solely discriminated patients in the high CSS group from the low CSS group for risk of CM (HR = 4.077, *p* = 0.002) and MACCE (HR = 1.753, *p* = 0.002) (**Supplementary Table 3**). The independent predictors of clinical results as per multivariate Cox proportional hazards regression analysis have been illustrated in Fig. 3 and **Supplementary Fig. 1**. After adjusting for confounding factors such as hypertension, diabetes, dyslipidemia, and New York Heart Association (NYHA) Grade, the CSS served as a predictor independently for ACM, CM, unplanned revascularization, MI, stroke, and MACCE (*p* < 0.05).

### 3.4 Predictive Performance of CSS Compared to SS and ${\mathrm{ACEF}}_{\text{CrCL}}$

**Fig. 4.**
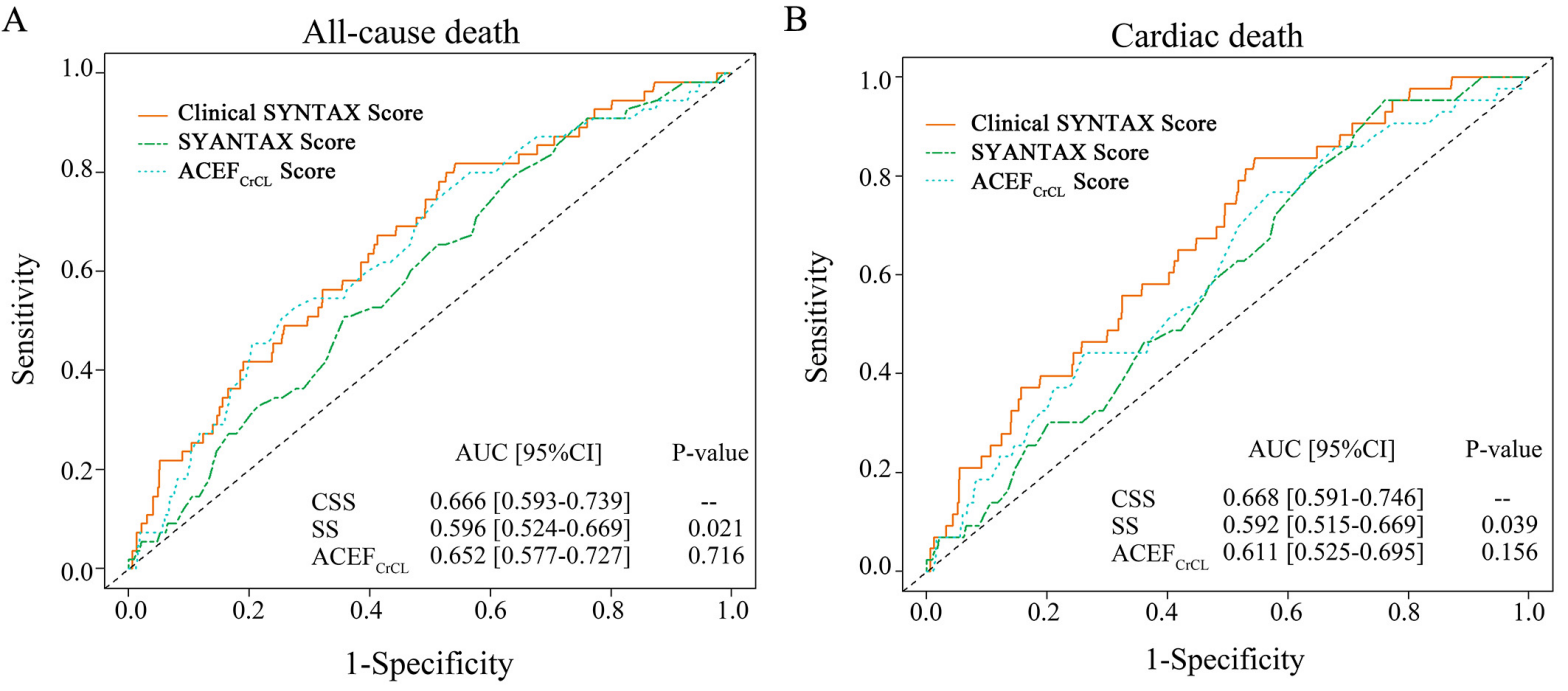
**ROC curves for all-cause mortality (A) and cardiac mortality (B) at median 3-year follow-up for the SS, modified ACEF score, and CSS**. AUC, area under the curve; SS, SYNTAX score; CSS, clinical SYNTAX score; ${\mathrm{ACEF}}_{\text{CrCl}}$ score, age/ejection fraction + 1 for each 10 mL the creatinine clearance <60 mL/min per 1.73 $m^{2}$.

Fig. 4 depicts the ROC curves for ACM and CM considering the SS, ${\mathrm{ACEF}}_{\text{CrCL}}$, and CSS. The C-statistics of CSS, SS, and ${\mathrm{ACEF}}_{\text{CrCL}}$ were 0.666, 0.596, and 0.652 for ACM, and 0.668, 0.592, and 0.611 for CM, respectively. The CSS demonstrated superior predictive capability compared to SS for median 3-year ACM (*p* = 0.021) and CM (*p* = 0.039).

### 3.5 Calibration Plots of the CSS

**Fig. 5.**
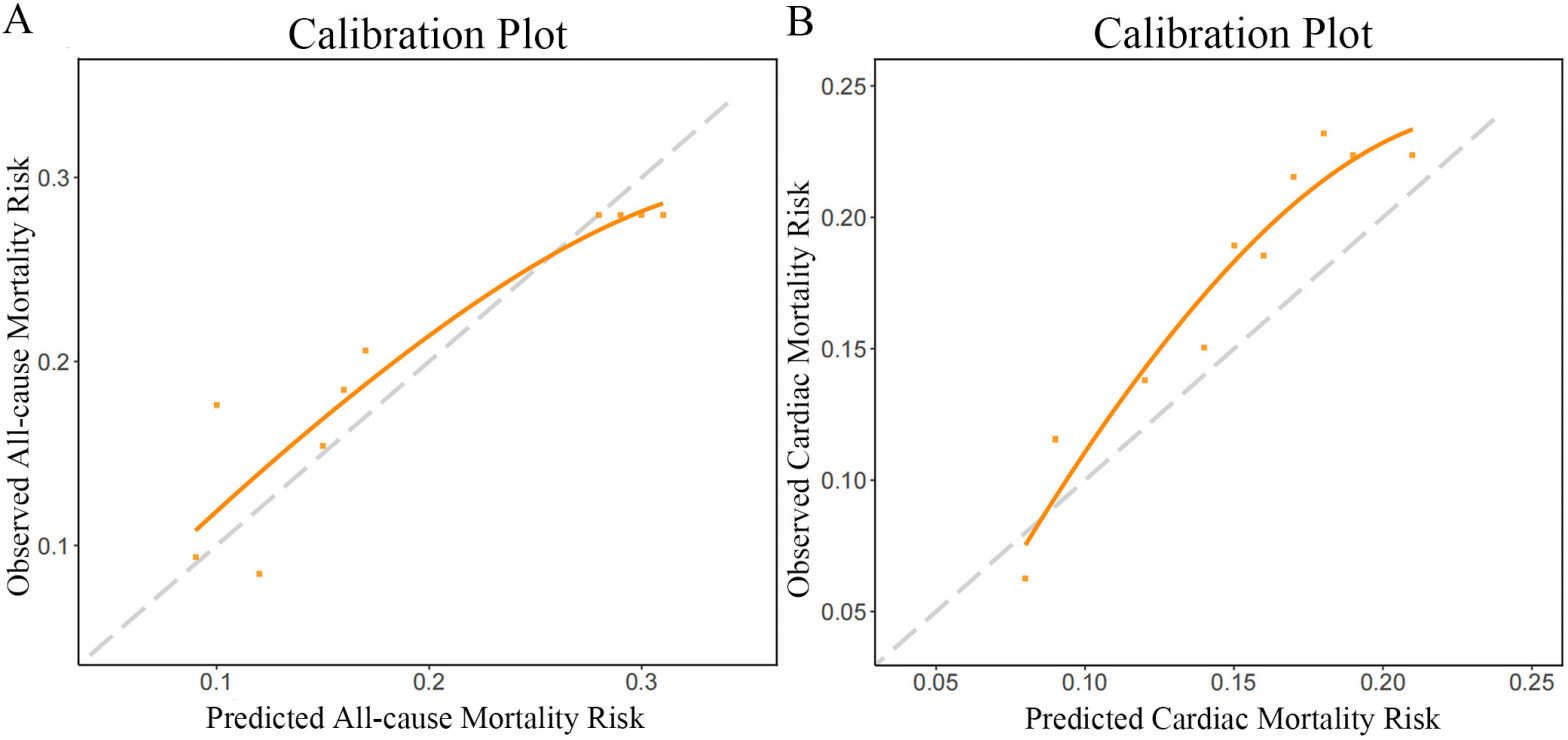
**Calibration curves for all-cause mortality (A) and cardiac mortality (B) at median 3-year follow-up for CSS**. CSS, clinical SYNTAX score.

The calibration curves of the CSS, assessing the probability of ACM and CM, demonstrated a good agreement between prediction and observation (Fig. 5). The Hosmer-Lemeshow tests yielded non-significant statistics implying that there was no departure from perfect fit for ACM (*p* = 0.632), CM (*p* = 0.444), MI (*p* = 0.485), unplanned revascularization (*p* = 0.734), and MACCE (*p* = 0.293). However, the Hosmer-Lemeshow test for stroke was statistically significant (*p* = 0.024) (Table 2).

**Table 2.** **Prognostic value of CSS for clinical outcomes at median 3-year follow-up**.

Variables	HR (95% CI) ^a^	*p*-value	AUC (95% CI) ^b^	H- L χ^2^
				(*p*-value) ^b^
All-cause mortality	1.017 (1.009–1.025)	<0.001	0.666 (0.593–0.739)	6.140 (0.632)
Cardiac mortality	1.016 (1.007–1.025)	0.001	0.668 (0.591–0.746)	7.889 (0.444)
Myocardial infarction	1.013 (1.004–1.023)	0.007	0.651 (0.541–0.762)	7.490 (0.485)
Unplanned revascularization	1.011 (1.003–1.018)	0.006	0.625 (0.562–0.688)	5.221 (0.734)
Stroke	1.015 (1.007–1.022)	<0.001	0.656 (0.590–0.723)	17.594 (0.024)
MACCE	1.010 (1.005–1.016)	<0.001	0.594 (0.565–0.628)	9.618 (0.290)

CI, confidence interval; AUC, area under the curve; CSS, clinical SYNTAX Score (continuous variable); H-L, Hosmer-Lemeshow; HR, hazard ratio; MACCE, major adverse cardiovascular and cerebrovascular events. ^a^ After adjustment for confounding factors. ^b^ For the entire model.

## 4. Discussion

This research verified the prognostic significance of CSS in patients with left main and/or three-vessel CAD and CRI undergoing PCI. The primary finding of the current research was as follows: following PCI with left main and/or three-vessel CAD and CRI patients, the CSS exhibited superior predictive performance compared to the SS in relation to ACM and CM. Additionally, the CSS served as an independent predictor of long-term ACM, CM, MI, stroke, unplanned revascularization, and MACCE.

Chronic kidney disease (CKD) is a considerable concern of public health worldwide [14]. It is believed to have a prevalence of 14% in the United States [15]. Over the period of 1990 to 2016, the global incidence, prevalence, deaths, and DALYs related to CKD have increased by 89%, 87%, 98%, and 62%, respectively [14]. The National Institute of Diabetes and Digestive and Kidney Diseases (NIDDK) established the Chronic Renal Insufficiency Cohort (CRIC) study, a multicentric prospective cohort research [16]. It has been revealed that CRI was significantly associated with left ventricular hypertrophy, heart failure [17], vascular stiffness, coronary artery calcification [18, 19], and adverse cardiovascular events [20]. Furthermore, prior investigations have revealed that CRI is associated with lower surgical success rates, more severe complications, increased risk of restenosis, recurrent MI, and stent thrombosis [3].

Within daily clinical practice, the stratification of risk and risk-benefit assessment following PCI hold immense importance. The SS, derived from the complexity and severity of CAD, has been demonstrated to be a valuable tool for stratifying complex CAD patients to assist in decisions of revascularization [4, 5]. Additional studies have substantiated its ability to predict clinical results following PCI in diverse clinical settings [21, 22]. However, SS’s prognostic significance was questioned for its accuracy and specificity, owing to its lack of incorporation of clinical characteristics that influence clinical outcomes [9]. The ROC analysis in the current research indicated a modest predictive value of SS for median 3-year ACM and CM. In addition, the C-statistics for ACM and CM were 0.597 and 0.592, respectively, and these values were insufficient to serve as a reference for clinical practice.

Earlier research has highlighted that scoring systems which incorporate anatomic and clinical variables are superior to angiographic SS [23]. The ACEF score, established with only age, LVEF, and serum creatinine values, has validated its comparability to complex scores, like the European System for Cardiac Operative Risk Evaluation (EUROSCORE) which included 17 clinical variables [24]. The CSS, incorporating both anatomical features and clinical variables (as with the ${\mathrm{ACEF}}_{\text{CrCL}}$ score), has been proven to be a convenient and straightforward predictive tool for predicting clinical results [8, 9]. The incremental prognostic value of the CSS was initially unveiled in the ARTS-II study involving 512 patients, as observed by Garg *et al*. [8]. The C-statistics for the CSS, SS, and ${\mathrm{ACEF}}_{\text{CrCL}}$ scores for 5-year MACCE were 0.62, 0.59, and 0.57, and were 0.69, 0.62, and 0.65, respectively, for 5-year mortality. The prognosis predictive ability of CSS for clinical outcomes for a long time in patients enduring PCI was confirmed by Girasis and his colleagues. The respective AUCs for SS and CSS for 5-year major adverse cardiac events (MACE) were 0.61 and 0.62, for 5-year ACM were 0.58 and 0.66, and for 5-year CM were 0.63 and 0.72 [9]. Capodanno *et al*. [25] demonstrated that CSS exhibited a superior discriminatory ability in assessing the risk of CM in patients with left main CAD post PCI, when compared against SS and EuroSCORE. For a 2-year CM, the CSS was demonstrated to have a satisfactory predictive capacity (AUC: 0.762). Recently, He *et al*. [26] validated the predictive value of CSS in acute coronary syndrome patients following PCI on 2-year clinical results. They revealed that CSS had a significantly superior performance for 2-year CM (AUC: 0.74 vs. 0.62, *p* < 0.001) but not for MACE (AUC: 0.60 vs. 0.59, *p* = 0.290) compared with baseline SS.

This study marks the first validation of CSS’s predictive significance in terms of median 3-year outcomes for patients with complex CAD and CRI following PCI. As per the findings of this research, CSS exhibited superior accuracy in predicting ACM (AUC: 0.666 vs. 0.597, *p* = 0.018) and CM (AUC: 0.668 vs. 0.592, *p* = 0.035) in comparison to SS. The performance of CSS for predicting ACM resembled the results obtained by Garg and Girasis [8]. However, the predictive ability of CSS for CM was notably lower compared with the studies conducted by Capodanno *et al*. [25] and He *et al*. [26] (AUC: 0.668 vs. 0.762 or 0.740). A possible explanation for this difference could be the fact that the population and follow-up timing in these studies were different.

## 5. Limitations

The current research has several limitations. First, owing to the post-hoc nature of the analysis, the findings should only be used to form hypotheses. Second, patients with prior PCI or CABG, prior MI, and a previous history of undergoing other cardiac surgery and malignant tumors were excluded from this research. Therefore, a selection bias might be present. Third, in this research, the fractional flow reserve (FFR) to determine the functional significance of coronary artery lesions was not used, as recommended by international guidelines in clinical practice [6]. Finally, this was a single-center, real-world study. To effectively understand individual performance with diverse risk models, further prospective, multicenter, and large-sample clinical studies should be conducted

## 6. Conclusions

The CSS significantly improved risk stratification for median 3-year ACM and CM in comparison with SS. Hence, this allowed for an individualized risk assessment in complex CAD and CRI patients following PCI.

## Data Availability

The authors are committed to providing raw data supporting the conclusions of this study. The detailed data related to the findings of this study are available from the corresponding author upon reasonable request.

## Supplementary Material

Supplementary material associated with this article can be found, in the online version, at https://doi.org/10.31083/j.rcm2501018.

## References

[1] Provenzano M, Coppolino G, Faga T, Garofalo C, Serra R, Andreucci M (2019). Epidemiology of Cardiovascular Risk in Chronic Kidney Disease Patients: the Real Silent Killer. *Reviews in Cardiovascular Medicine*.

[2] Vallianou NG, Mitesh S, Gkogkou A, Geladari E (2018). Chronic Kidney Disease and Cardiovascular Disease: is there any Relationship. *Current Cardiology Reviews*.

[3] Tsai TT, Messenger JC, Brennan JM, Patel UD, Dai D, Piana RN (2011). Safety and Efficacy of Drug-Eluting Stents in Older Patients with Chronic Kidney Disease. *Journal of the American College of Cardiology*.

[4] Sianos G, Morel MA, Kappetein AP, Morice MC, Colombo A, Dawkins K (2005). The SYNTAX Score: An Angiographic Tool Grading the Complexity of Coronary Artery Disease. *EuroIntervention*.

[5] Serruys P, Onuma Y, Garg S, Sarno G, van den Brand M, Kappetein A (2009). Assessment of the SYNTAX score in the Syntax Study. *EuroIntervention*.

[6] Sousa-Uva M, Neumann FJ, Ahlsson A, Alfonso F, Banning AP, Benedetto U (2019). 2018 ESC/EACTS Guidelines on Myocardial Revascularization. *European Journal of Cardio-Thoracic Surgery*.

[7] Farooq V, Vergouwe Y, Généreux P, Bourantas CV, Palmerini T, Caixeta A (2013). Prediction of 1-Year Mortality in Patients with Acute Coronary Syndromes Undergoing Percutaneous Coronary Intervention. *JACC: Cardiovascular Interventions*.

[8] Garg S, Sarno G, Garcia-Garcia HM, Girasis C, Wykrzykowska J, Dawkins KD (2010). A New Tool for the Risk Stratification of Patients with Complex Coronary Artery Disease. *Circulation: Cardiovascular Interventions*.

[9] Girasis C, Garg S, Räber L, Sarno G, Morel M, Garcia-Garcia HM (2011). SYNTAX score and Clinical SYNTAX score as Predictors of Very Long-term Clinical Outcomes in Patients Undergoing Percutaneous Coronary Interventions: a substudy of SIRolimus-eluting Stent Compared with pacliTAXel-eluting Stent for Coronary Revascularization (SIRTAX) Trial. *European Heart Journal*.

[10] Yan L, Li P, Wang Y, Han D, Li S, Jiang M (2021). The Incremental Prognostic Value of the Clinical Residual SYNTAX Score for Patients With Chronic Renal Insufficiency Undergoing Percutaneous Coronary Intervention. *Frontiers in Cardiovascular Medicine*.

[11] Yan L, Li P, Wang Y, Han D, Li S, Zhang J (2020). Impact of the Residual SYNTAX Score on Clinical Outcomes after Percutaneous Coronary Intervention for Patients with Chronic Renal Insufficiency. *Catheterization and Cardiovascular Interventions*.

[12] Thygesen K, Alpert JS, Jaffe AS, Chaitman BR, Bax JJ, Morrow DA (2019). Fourth universal definition of myocardial infarction (2018). *European Heart Journal*.

[13] Van Hoorde K, Vergouwe Y, Timmerman D, Van Huffel S, Steyerberg EW, Van Calster B (2014). Assessing Calibration of Multinomial Risk Prediction Models. *Statistics in Medicine*.

[14] Xie Y, Bowe B, Mokdad AH, Xian H, Yan Y, Li T (2018). Analysis of the Global Burden of Disease Study Highlights the Global, Regional, and National Trends of Chronic Kidney Disease Epidemiology from 1990 to 2016. *Kidney International*.

[15] Hannan M, Ansari S, Meza N, Anderson AH, Srivastava A, Waikar S (2021). Risk Factors for CKD Progression. *Clinical Journal of the American Society of Nephrology*.

[16] Feldman HI, Appel LJ, Chertow GM, Cifelli D, Cizman B, Daugirdas J (2003). The Chronic Renal Insufficiency Cohort (CRIC) Study. *Journal of the American Society of Nephrology*.

[17] Park M, Shlipak MG, Katz R, Agarwal S, Ix JH, Hsu C (2012). Subclinical Cardiac Abnormalities and Kidney Function Decline. *Clinical Journal of the American Society of Nephrology*.

[18] Budoff MJ, Rader DJ, Reilly MP, Mohler ER, Lash J, Yang W (2011). Relationship of Estimated GFR and Coronary Artery Calcification in the CRIC (Chronic Renal Insufficiency Cohort) Study. *American Journal of Kidney Diseases*.

[19] Townsend RR, Wimmer NJ, Chirinos JA, Parsa A, Weir M, Perumal K (2010). Aortic PWV in Chronic Kidney Disease: a CRIC Ancillary Study. *American Journal of Hypertension*.

[20] Denker M, Boyle S, Anderson AH, Appel LJ, Chen J, Fink JC (2015). Chronic Renal Insufficiency Cohort Study (CRIC). *Clinical Journal of the American Society of Nephrology*.

[21] Guedeney P, Barthélémy O, Zeitouni M, Hauguel-Moreau M, Hage G, Kerneis M (2020). Prognostic Value of SYNTAX Score in Patients with Infarct-Related Cardiogenic Shock. *JACC: Cardiovascular Interventions*.

[22] Xu M, Chen H, Li H (2022). The Association Between SYNTAX Score and Long-term Outcomes in Patients with Unstable Angina Pectoris: a Single-centre Retrospective Study. *BMC Cardiovascular Disorders*.

[23] Yadav M, Palmerini T, Caixeta A, Madhavan MV, Sanidas E, Kirtane AJ (2013). Prediction of Coronary Risk by SYNTAX and Derived Scores. *Journal of the American College of Cardiology*.

[24] Ranucci M, Castelvecchio S, Menicanti L, Frigiola A, Pelissero G (2009). Risk of Assessing Mortality Risk in Elective Cardiac Operations. *Circulation*.

[25] Capodanno D, Caggegi A, Miano M, Cincotta G, Dipasqua F, Giacchi G (2011). Global Risk Classification and Clinical SYNTAX (Synergy between Percutaneous Coronary Intervention with TAXUS and Cardiac Surgery) Score in Patients Undergoing Percutaneous or Surgical Left Main Revascularization. *JACC: Cardiovascular Interventions*.

[26] He C, Song Y, Wang C, Yao Y, Tang X, Zhao X (2017). Prognostic Value of the Clinical SYNTAX Score on 2-Year Outcomes in Patients with Acute Coronary Syndrome who Underwent Percutaneous Coronary Intervention. *The American Journal of Cardiology*.

