# Supplementary material for: Prognostic Capability of Clinical SYNTAX Score in Patients with Complex Coronary Artery Disease and Chronic Renal Insufficiency Undergoing Percutaneous Coronary Intervention
Source: Rev Cardiovasc Med. 2024 Jan 10;25(1):18. doi: 10.31083/j.rcm2501018 (PMC11262395; doi:10.31083/j.rcm2501018)
Supplement: Supplementary file 1 [file 2153-8174-25-1-018-s1.zip › 2153-8174-25-1-018-s1/Supplementary document.docx]

Supplementary Table 1. Lesions’ anatomical features and procedural details

|  | CSS < 18.0  (n = 321) | CSS < 18.0-28.3  (n = 317) | CSS > 28.3  (n = 324) | *p*-value |
| --- | --- | --- | --- | --- |
| Lesion anatomical characteristics |  |  |  |  |
| Heavy calcification | 10 (3.2) | 40 (12.5) | 89 (27.5) | < 0.001 |
| Tortuous | 10 (3.2) | 26 (8.3) | 41 (12.5) | < 0.001 |
| Bifurcation | 62 (19.4) | 106 (33.3) | 146 (45.0) | < 0.001 |
| Diffuse | 189 (58.9) | 224 (70.8) | 254 (78.3) | < 0.001 |
| Chronic total occlusions | 18 (5.6) | 61 (19.2) | 106 (32.5) | < 0.001 |
| Aorto-ostial lesion | 15 (4.8) | 10 (3.3) | 22 (6.7) | 0.151 |
| Thrombus | 21 (6.5) | 48 (15.0) | 43 (13.3) | 0.002 |
| Target lesion location |  |  |  |  |
| LM | 9 (2.8) | 15 (4.7) | 35 (10.8) | < 0.001 |
| LAD | 144 (44.9) | 190 (59.9) | 177 (54.6) | < 0.001 |
| LCX | 135 (42.1) | 103 (32.5) | 97 (29.9) | 0.030 |
| RCA | 168 (52.3) | 163 (51.4) | 150 (46.3) | 0.255 |
| Procedural characteristics |  |  |  |  |
| The total length of the stent, mm | 48 (30-67) | 56 (35-84) | 52 (33-72) | 0.014 |
| Stent length > 100mm | 25 (7.8) | 43 (13.6) | 37 (11.4) | 0.061 |
| Mean stent diameter, mm | 3.0 (2.75-3.25) | 3.0 (2.75-3.25) | 3.0 (2.75-3.25) | 0.875 |
| Minimum stent diameter, mm | 2.75 (2.50-3.0) | 2.75 (2.5-3.0) | 2.75 (2.5-3.0) | 0.502 |
| Maximum stent diameter, mm | 3.0 (2.75-3.5) | 3.0 (2.75-3.5) | 3.0 (2.75-3.5) | 0.966 |
| SYNTAX score |  |  |  | < 0.001 |
| SS < 22 | 318 (99.1) | 253 (79.8) | 119 (36.7) |  |
| SS < 22-32 | 3 (0.9) | 63 (19.9) | 154 (47.5) |  |
| SS > 32 | 0 (0) | 1 (0.3) | 51 (15.7) |  |
| SYNTAX score | 13.0 (11.0-15.0) | 19.0 (16.0-22.0) | 25.0 (20.6-28.9) | < 0.001 |
| Clinical SYNTAX score | 14.2 (11.7-16.0) | 22.7 (20.1-25.1) | 38.6 (31.8-53.0) | < 0.001 |

*Values are mean ± SD, median (IQR), or n (%). LM: left main; LAD: left anterior descending artery; LCX: left circumflex; RCA: right coronary artery*

Supplementary Table 2. Five-year cumulative incidence of adverse events

|  | CSS < 18.0  (n = 321)  (a) | CSS < 18.0-28.3  (n = 317)  (b) | CSS > 28.3  (n = 324)  (c) |  | *p*-value | | | |
| --- | --- | --- | --- | --- | --- | --- | --- | --- |
|  |  |  |  |  | Trend | a vs. b* | b vs. c* | a vs. c* |
| All-cause mortality | 3.6% (9) | 6.6% (14) | 19.4% (31) |  | 0.001 | 0.206 | 0.017 | <0.001 |
| Cardiac mortality | 3.2% (6) | 5.1% (13) | 15.6% (24) |  | 0.003 | 0.098 | 0.071 | 0.001 |
| Myocardial infarction | 3.8% (5) | 7.4% (10) | 13.3% (23) |  | 0.001 | 0.194 | 0.025 | 0.001 |
| Stroke | 7.3% (13) | 9.1% (19) | 19.5% (41) |  | <0.001 | 0.225 | 0.005 | <0.001 |
| Unplanned  revascularization | 7.2% (16) | 12.8% (24) | 19.5% (40) |  | 0.003 | 0.161 | 0.064 | 0.001 |
| MACCE | 20.0% (49) | 29.0% (59) | 33.8% (84) |  | 0.002 | 0.225 | 0.051 | 0.001 |

*Note: Event rates are Kaplan-Meier estimates, % (n). *Adjusted significance level is 0.017.*

*MACCE: Major adverse cardiovascular and cerebrovascular events.*

Supplementary Table 3. Univariable Cox regression analysis of long-term outcomes

|  | All-cause mortality | | Cardiac mortality | | MACCE | |
| --- | --- | --- | --- | --- | --- | --- |
|  | HR(95%CI) | P-value | HR(95%CI) | P-value | HR(95%CI) | P-value |
| CSS | 1.907(1.335-2.726) | <0.001 | 1.978(1.316-2.973) | 0.001 | 1.330(1.116-1.585) | 0.001 |
| Low vs. Mid | 1.679(0.734-3.837) | 0.219 | 2.205(0.838-5.803) | 0.109 | 1.261(0.864-1.843) | 0.229 |
| Low vs. High | 3.485(1.659-7.320) | 0.001 | 4.077(1.666-9.975) | 0.002 | 1.753(1.232-2.494) | 0.002 |
| Mid vs. High | 2.075(1.121-3.846) | 0.020 | 1.848(0.941-3.636) | 0.074 | 1.389(0.996-1.938) | 0.053 |
| Age | 1.049(1.013-1.086) | 0.007 | 1.033(0.994-1.073) | 0.0103 | 1.010(0.992-1.028) | 0.269 |
| Female | 0.840(0.488-1.448) | 0.531 | 0.892(0.484-1.645) | 0.715 | 0.857(0.641-1.145) | 0.297 |
| BMI | 1.046(0.962-1.137) | 0.291 | 1.068(0.972-1.173) | 0.172 | 1.031(0.986-1.079) | 0.174 |
| eGFR | 0.971(0.957-0.985) | <0.001 | 0.965(0.950-0.981) | <0.001 | 0.979(0.971-0.988) | <0.001 |
| Hypertension | 0.519(0.287-0.940) | 0.030 | 0.821(0.438-1.538) | 0.054 | 1.280(0.927-1.767) | 0.034 |
| Diabetes | 1.931(1.119-3.329) | 0.018 | 2.715(1.488-4.950) | 0.001 | 1.516(1.123-2.048) | 0.007 |
| Hyperlipidemia | 0.715(0.415-1.232) | 0.227 | 0.478(0.241-0.950) | 0.035 | 0.999(0.749-1.332) | 0.094 |
| Previous Smoke | 1.401(0.661-2.967) | 0.379 | 1.069(0.421-2.718) | 0.888 | 0.892(0.561-1.417) | 0.628 |
| Previous Stroke | 0.438(0.137-1.403) | 0.165 | 0.572(0.177-1.850) | 0.351 | 1.175(0.771-1.789) | 0.453 |
| COPD | 0.049(0.000-6.184) | 0.531 | 0.049(0.00-18.154) | 0.573 | 1.012(0.323-3.167) | 0.984 |
| NYHA Grade | 2.261(1.429-3.576) | <0.001 | 2.594(1.581-4.257) | <0.001 | 1.598(1.195-2.138) | 0.002 |
| SYNTAX Score | 1.046(1.010-1.083) | 0.011 | 1.045(1.005-1.086) | 0.028 | 1.030(1.010-1.050) | 0.003 |
|  | Stroke | | Unplanned revascularization | | Myocardial infarction | |
|  | HR(95%CI) | P-value | HR(95%CI) | P-value | HR(95%CI) | P-value |
| CSS | 1.853(1.364-2.518) | <0.001 | 1.586(1.197-2.101) | 0.001 | 2.186(1.396-3.424) | 0.001 |
| Low vs. Mid | 1.504(0.742-3.045) | 0.257 | 1.559(0.828-2.935) | 0.169 | 2.026(0.692-5.928) | 0.198 |
| Low vs. High | 3.219(1.725-6.007) | <0.001 | 2.504(1.402-4.471) | 0.002 | 4.636(1.762-12.197) | 0.002 |
| Mid vs. High | 2.141(1.242-3.690) | 0.006 | 1.605(0.968-2.667) | 0.066 | 2.288(1.089-4.808) | 0.029 |
| Age | 1.019(0.989-1.049) | 0.213 | 1.004(0.977-1.031) | 0.787 | 1.064(1.019-1.110) | 0.004 |
| Female | 0.722(0.447-1.165) | 0.182 | 0.999(0.641-1.557) | 0.998 | 1.094(0.577-2.074) | 0.783 |
| BMI | 1.006(0.935-1.082) | 0.876 | 1.007(0.939-1.080) | 0.847 | 0.967(0.874-1.071) | 0.524 |
| eGFR | 0.972(0.960-0.985) | <0.001 | 0.985(0.971-0.999) | 0.031 | 0.973(0.956-0.991) | 0.003 |
| Hypertension | 1.615(0.927-2.812) | 0.091 | 1.425(0.851-2.386) | 0.178 | 3.837(1.361-10.816) | 0.011 |
| Diabetes | 1.597(0.985-2.590) | 0.058 | 1.910(1.218-2.996) | 0.005 | 2.536(1.336-4.812) | 0.004 |
| Hyperlipidemia | 1.221(0.771-1.935) | 0.394 | 1.038(0.666-1.618) | 0.086 | 0.793(0.410-1.534) | 0.490 |
| Previous Smoke | 1.134(0.564-2.278) | 0.725 | 0.872(0.420-1.811) | 0.714 | 0.968(0.343-2.729) | 0.951 |
| Previous Stroke | 1.700(0.933-3.099) | 0.083 | 1.071(0.552-2.079) | 0.184 | 1.681(0.739-3.823) | 0.215 |
| COPD | 1.882(0.462-7.675) | 0.378 | 1.577(0.387-6.421) | 0.525 | 1.662(0.228-12.123) | 0.616 |
| NYHA Grade | 1.73 0(1.096-2.731) | 0.019 | 1.538(0.975-2.426) | 0.064 | 2.002(1.115-3.595) | 0.020 |
| SYNTAX Score | 1.054(1.024-1.086) | <0.001 | 1.049(1.020-1.079) | 0.001 | 1.063(1.020-1.107) | 0.003 |

*Values are mean ± SD, median (IQR), or n (%). CSS：Clinical SYNTAX Score;BMI: body mass index;COPD: chronic obstructive pulmonary diseas*

**Supplementary Fig. 1.** Multivariate Cox proportional hazards regression for myocardial infarction, stroke and unplanned revascularization.

*
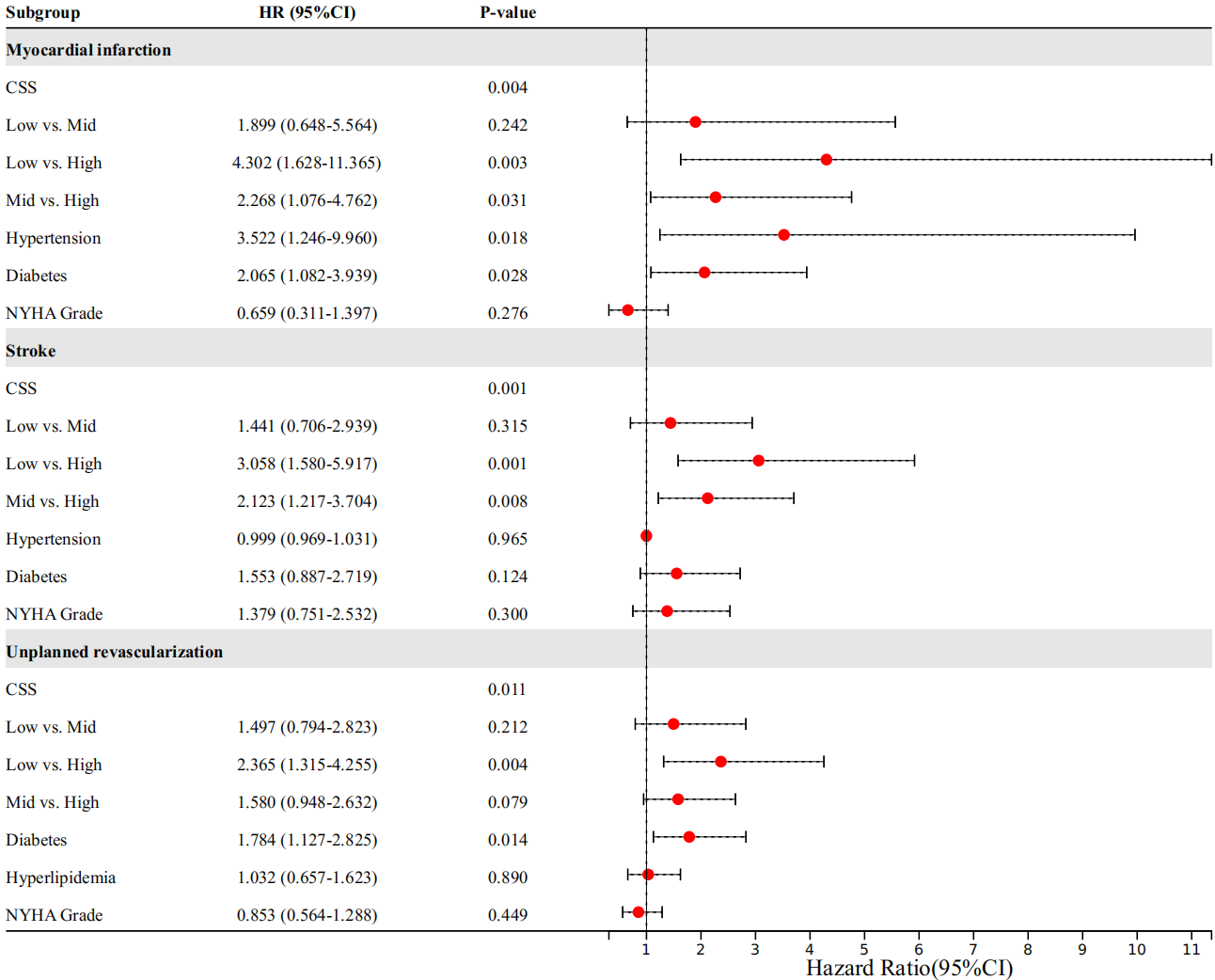
*
