# Supplementary figures and images for: Prognostic Capability of Clinical SYNTAX Score in Patients with Complex Coronary Artery Disease and Chronic Renal Insufficiency Undergoing Percutaneous Coronary Intervention
Source: Rev Cardiovasc Med. 2024 Jan 10;25(1):18. doi: 10.31083/j.rcm2501018 (PMC11262395; doi:10.31083/j.rcm2501018)

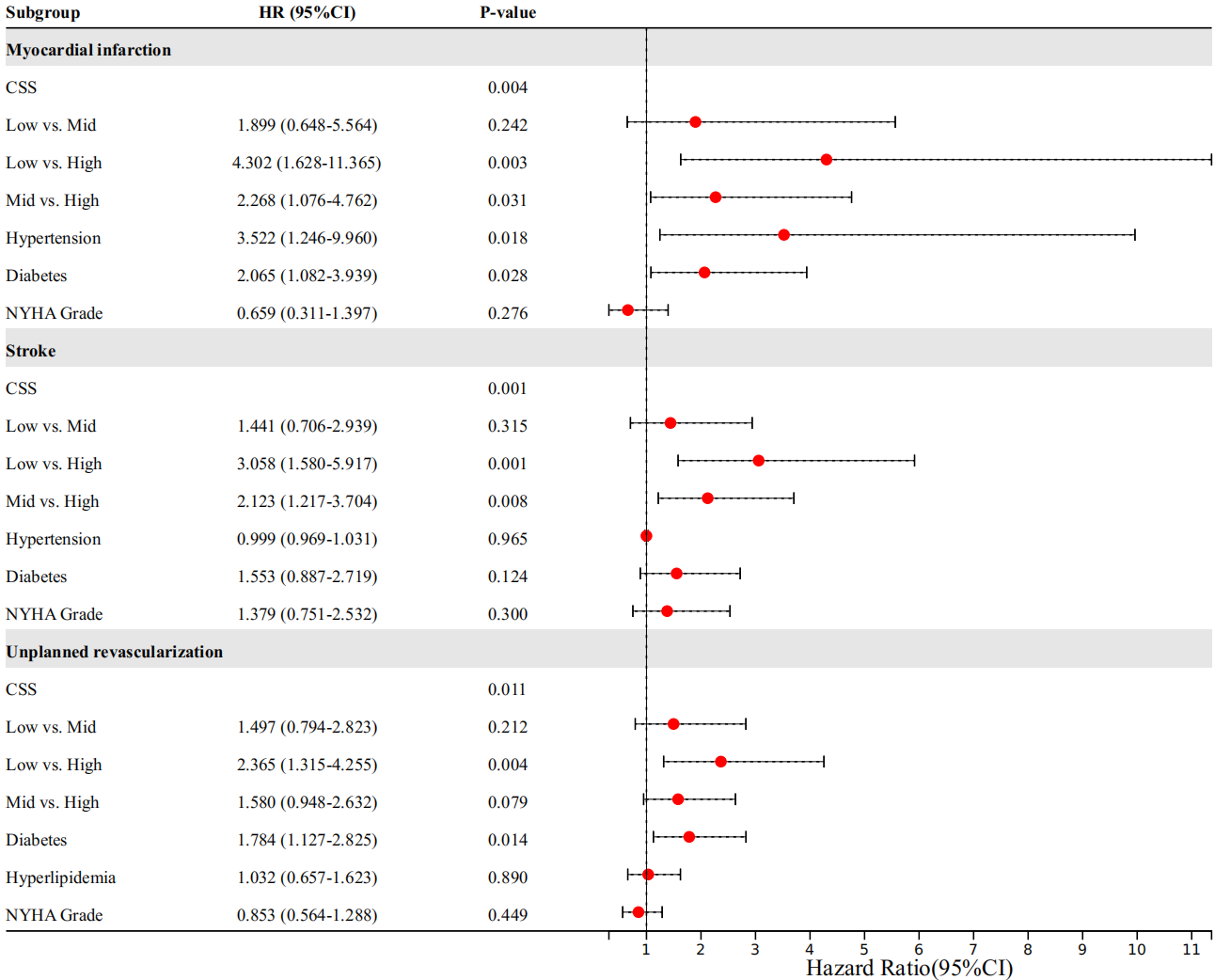

Supplement: Supplementary file 1 [file 2153-8174-25-1-018-s1.zip › 2153-8174-25-1-018-s1/Supplementary Figure. Forest plot for clinical outcomes according to multivariate Cox regression analysis..tiff]
